# Hypnotism and Crime

**Published:** 1895-05

**Authors:** 


### HYPNOTISM AND CRIME.

The Kansas courts have lately rendered decisions *in re* hypnotism which we believe to be setting an exceedingly bad precedent. It appears that Thomas Patton was shot and killed about a year ago by one McDonald. The latter, on trial, admitted the killing, but alleged that the murder was done while he was under the hypnotic influence of Anderson Gray; that he was therefore not responsible.

Gray was not present at the killing. McDonald was acquitted and Gray convicted. The finding of the first court was sustained on appeal to the Supreme Court (April 6).

So far as we know, this is the first time that this matter has been adjudicated in this country. About two years ago an important case in Paris was decided in the opposite manner. The precedent thus established will be eagerly seized as a defense by future criminals. The phenomena of hypnotism are too complex and too little understood for the ends of justice to be defeated by this sort of defense. If these decisions are to be followed in other courts, we may expect to see the hypnotic irresponsibility plea become unfortunately too fashionable.

